# Accuracy of diagnostic tests for perilymphatic fistula: protocol for a systematic review

**DOI:** 10.3389/fneur.2024.1500780

**Published:** 2024-11-18

**Authors:** Maksym Situkho, Tetsuo Ikezono, Marte Myhrum, Arne Kirkhorn Rødvik, Yukihide Maeda, Han Matsuda, Masafumi Sawada, Greg Eigner Jablonski

**Affiliations:** ^1^Department of Otorhinolaryngology and Head and Neck Surgery, Oslo University Hospital, Oslo, Norway; ^2^Institute for Clinical Medicine, Faculty of Medicine, University of Oslo, Oslo, Norway; ^3^Department of Otorhinolaryngology, Faculty of Medicine, Saitama Medical University, Saitama, Japan

**Keywords:** perilymphatic fistula, diagnostic test accuracy, systematic review, biomarkers, vestibular disorders

## Abstract

Perilymphatic fistula (PLF) is a challenging inner ear condition, characterized by the abnormal connection between inner and middle ear and subsequent leakage of inner ear liquid (perilymph) into the middle ear. Early surgical intervention for PLF might lead to favorable audiological and vestibular outcomes. The conventional reference standard of PLF detection is the intraoperative visualization of perilymph leakage during explorative tympanotomy or endoscopic inspection; the specificity and sensitivity of this method is unknown. Systematic reviews assessing the accuracy of diagnostic tests for PLF are not available.

**Objective:**

To systematically review the literature for qualitative evidence exploring the diagnostic tests for the PLF diagnosis. The proposed systematic review will answer the following question: What is the accuracy of diagnostic tests in detecting perilymphatic fistula in humans?

**Methods:**

Eligibility criteria: original peer-reviewed articles regarding studies on humans of any age containing data with diagnostic test accuracy estimation (sensitivity and specificity) for PLF diagnosis or in which diagnostic test accuracy could be calculated based on data provided, without language, study design or study date limits. MEDLINE, EMBASE, the Cochrane Central Register of Controlled Trials, Scopus, and Web of Science will be searched for eligible articles. Additional manual searches will be performed. Covidence software will be used for title and abstract screening, full text review and data extraction. The risk of bias assessment will be conducted using the Quality Assessment of Diagnostic Test Accuracy 2 (QUADAS-2) tool. If two or more high-quality articles evaluating the same diagnostic test will be identified, their findings will be quantitatively synthesized; if a quantitative synthesis is not feasible, the results will be described in a narrative summary. Grading of Recommendations, Assessment, Development, and Evaluations (GRADE) framework will be used to evaluate evidence strength. Study funded by Oslo University Hospital.

## 1 Introduction

### 1.1 Background

Perilymphatic fistula (PLF) is a difficult to diagnose inner ear condition, which is characterized by the abnormal connection between inner and middle ear and subsequent leakage of inner ear liquid (perilymph) into the middle ear (1, 2). The primary manifestations of the PLF are sudden or progressive, fluctuating sensorineural hearing loss or deafness and/or vestibular symptoms ranging from dizziness to rotatory vertigo (3).

Several diagnostic criteria were published previously (4–6); the recent one is published from a Japanese PLF research group (7). However, due to the lack of internationally accepted diagnostic criteria and the difficulty of making a definitive diagnosis, the epidemiology of PLF needs to be clarified. The heterogeneity of the causing factors adds to the difficulty of disease prevalence and incidence measures (2, 8). It has been reported that PLF was intraoperatively diagnosed in 24% of patients with severe to profound sudden sensorineural hearing loss, not responding to high doses of steroids (9).

PLF can be caused by a wide range of factors, such as head trauma (10–12), changes in air pressure (e.g., during air travel) (13–16), barotrauma (5), chronic ear infections (17, 18), congenital abnormalities (19), or as a result of an iatrogenic injury during cochlear implantation or stapes surgery (20, 21). Individuals with occupations or activities involving exposure to sudden pressure changes or repeated physical strain may be at higher risk, especially divers and deployed military personnel (22–24).

Early diagnosis and surgical intervention for PLF can lead to favorable audiological and vestibular outcomes (1, 25). However, the prognosis varies among individuals, and not all patients experience complete resolution of symptoms. Improvement in vestibular symptoms tends to be more significant than auditory symptoms (3).

The diagnosis of perilymphatic fistula (PLF) is challenging and depends on the integration of clinical evaluations and functional tests. Investigations include comprehensive audio-vestibular testing, such as pure tone, speech, and positional audiometry (7, 26), electro- and videonystagmography (27), vestibular evoked myogenic potentials (28), electrocochleography (29), video Head Impulse Testing (30), posturography (31), MRI and high-resolution computed tomography (1, 5, 32). Biomarkers, such as beta-2 transferrin, beta-trace protein, and Cochlin-tomoprotein, are also being used in diagnosis (33–37).

The conventional reference standard of PLF detection is the intraoperative visualization of perilymph leakage during explorative tympanotomy or endoscopic inspection (9, 38, 39). The reference standard is far from being perfect; the specificity and sensitivity of this method is unknown (22). Direct visualization of the round and oval windows is not always possible, and the diagnosis cannot be excluded in case of the absence of perilymph leakage. The presence of transparent fluid collection in the round and oval window niches may not necessarily represent perilymph, as tissue fluid, local anesthetic, and irrigation fluid can all be mistaken for perilymph (1, 40, 41).

### 1.2 Relevance

The accuracy of diagnostic tests for PLF significantly affects individual health outcomes. Early and precise diagnosis facilitates appropriate treatment, potentially improving auditory and vestibular functions, and enhancing the quality of life of affected individuals.

Healthcare professionals could be more certain in selecting appropriate diagnostic methods for suspected PLF cases, thereby providing better patient management.

Accurate diagnostic tests for PLF are important for public health, as they enable awareness and early symptom recognition. This can prevent treatment delays, reduce PLF burden, and enhance public health outcomes.

This review’s evidence-based recommendations on PLF diagnostic tests can guide healthcare and reimbursement policies, guidelines, and resource allocation.

The review will also have strong implications for future PLF research. Identifying promising diagnostic tests can guide new studies, including the development of new diagnostic criteria, validation of existing tests in different cohorts, and biomarker exploration. This can advance diagnostic accuracy, deepen the understanding of PLF pathophysiology, and promote targeted treatment strategies. Moreover, comprehensive analysis of bias sources in previous studies, as provided by systematic reviews, can pave the way for new high-quality research.

### 1.3 Rationale

There are two existing systematic reviews related to PLF, but they do not specifically address the accuracy of diagnostic tests (42, 43). The first systematic review focuses on the presentation, management, and hearing outcomes of labyrinthine fistula secondary to cholesteatoma, while the second review explores the association between sneezing and perilymphatic fistula of the round window. Thus, existing systematic reviews on PLF offer valuable insights but do not address diagnostic test accuracy, leaving the current evidence insufficient to answer this question.

### 1.4 Objective

Our study’s objective is to systematically review the literature for qualitative evidence exploring the diagnostic tests for the PLF diagnosis. PIT (population, index tests, and target condition) methodology was used to define the research question (44).

The proposed systematic review will answer the following question:

What is the accuracy of diagnostic tests in detecting perilymphatic fistula in humans?

What are the PIT components of the review question/objective?

P (Population) – humans.

I (Index tests) – all available diagnostic tests.

T (target condition) – perilymphatic fistula.

## 2 Methods

### 2.1 Protocol development

The protocol has been developed using the Cochrane handbook for reviews of diagnostic test accuracy and the Preferred Reporting Items for a Systematic Review and Meta-analysis of Diagnostic Test Accuracy Studies (PRISMA-DTA) statement (45, 46). This protocol is reported in line with the PRISMA statement for review protocols (PRISMA-P), which is attached in Supplementary Table S1 (47).

### 2.2 Eligibility criteria

Studies on humans of any age containing data with diagnostic test accuracy estimation (sensitivity and specificity) for PLF diagnosis.

Studies on humans of any age on PLF, in which diagnostic test accuracy could be calculated based on data provided in the article.

Original peer-reviewed articles will be included.

No language limits will be imposed.

No study design restriction will be imposed.

### 2.3 Information sources

For the identification of relevant studies, we will use following electronic databases: MEDLINE (Ovid interface, 1948 onwards), EMBASE (Ovid interface, 1980 onwards), the Cochrane Central Register of Controlled Trials (CENTRAL, Wiley interface, latest issue), Scopus, and Web of Science. In addition, the reference lists of the included studies and relevant reviews will be manually examined.

After the completion of the search, a bibliography of the identified studies will be distributed among the research team and top experts in the field of perilymphatic fistula for review.

### 2.4 Search strategy

Two factors are important to consider in the context of our review search strategy. First, diagnostic test accuracy studies are often poorly reported in the titles and abstracts. Use of search filters to identify diagnostic accuracy studies might lead to missing relevant studies (48). Second, PLF is a rare condition and the amount of publication regarding it is limited. Therefore, our search strategy was fitted to identify as many studies considering the target condition as possible.

The strategy (Supplementary Table S2) was built with an expert in systematic searches from The Library of Medicine and Science, University of Oslo library.

Search results will be deduplicated using EndNote reference management software (Clarivate, London, United Kingdom) and then uploaded to the specialized online review tool, Covidence (Veritas Health Innovation, Melbourne, Australia). Two investigators will screen the titles and abstracts. Any disagreements will be resolved by consensus decision. Priority screening with machine learning to focus early screening effort on most relevant records will be utilized. If no consensus is achieved, a third investigator will be invited to resolve the disagreement. Two authors will independently conduct full text screening on the chosen articles. Subsequently, the selected articles will proceed to data extraction.

### 2.5 Data extraction

A structured data extraction form will be developed from scratch in the Covidence software, containing all relevant data fields based on the key data elements identified. The form will be designed to capture study characteristics, participant characteristics, details of the index test and reference standards, diagnostic accuracy measures, and other relevant outcomes.

Study characteristics:

- Author(s) and year of publication
- Dates of data collection of the study (if available)
- Study design (e.g., prospective cohort, case–control, diagnostic accuracy study)
- Country or setting where the study was conducted
- Duration of the study, if applicable

Participant characteristics:

- Total number of participants included in the study
- Characteristics of the study population (e.g., age, gender, and clinical presentation)
- Inclusion and exclusion criteria for participant selection
- Reason for the referral for the index test (symptoms, other disease, etc.)

Index test information:

- Description of the index test(s) used to diagnose PLF
- Type of index test (e.g., clinical, instrumental, biomarker, imaging)
- Details on the procedure and protocol of the index test (time between index test and reference standard)
- Threshold(s) used for dichotomous interpretation of the index test results

Reference standard information:

- Description of the reference standard(s) used to confirm or exclude PLF
- Details on the procedure and protocol of the reference standard
- Threshold(s) used for dichotomous interpretation of the reference standard results

Diagnostic accuracy measures:

- Sensitivity: The proportion of true positive results among individuals with PLF
- Specificity: The proportion of true negative results among individuals without PLF
- 95% confidence intervals or standard errors for the above measures (if available)
- If sensitivity/specificity is not calculated, True Positive/True Negative/False Positive/False Negative cases data will be retrieved (using 2*2 table)

The data extraction form will be pilot-tested on a subset of included studies to ensure that it captures all necessary information and is easy to use. Necessary revisions will be made based on feedback from the pilot testing.

A narrative summary of the key findings extracted from the included studies will be presented, highlighting important trends, variations, or inconsistencies in the data.

### 2.6 Assessment of the risk of bias

The risk of bias assessment for individual studies will be conducted using the Quality Assessment of Diagnostic Test Accuracy 2 (QUADAS-2) tool, which is designed to evaluate the quality of primary diagnostic accuracy studies, and consists of four key domains - patient selection, index test, reference standard, and flow and timing (49). QUADAS-2 checklist is provided in Supplementary Table S3. The assessment will be carried out independently by two reviewers. Any disagreements between the two reviewers will be resolved through discussion and consensus. If consensus cannot be reached, a third reviewer will be consulted. The information obtained from the assessment will be provided in a narrative summary and displayed in a table.

Articles with high risk of bias may be excluded from the data analysis if studies with less risk of bias provide similar information or when the extent of the bias could significantly distort the overall results.

### 2.7 Data synthesis

If two or more articles of appropriate quality evaluating the same diagnostic test are identified, their findings will be quantitatively synthesized into an analysis of sensitivity and specificity (45). If quantitative synthesis is not appropriate, data will be organized into tabular forms and explained in a detailed descriptive summary.

Based on the analysis of potentially relevant articles, the review will investigate two distinct study categories in perilymphatic fistula research.

1. Diagnostic studies in which explorative tympanotomy and perilymph leakage visualization were used as reference standard and performed after the index test in patients with suspected PLF. Data synthesis will consider the limitations of the reference standard. To address the imperfect reference standard and heterogeneity of index tests, a Bayesian methodology can be employed (50).
2. Diagnostic studies with perilymph leakage occurring during surgery, such as cochlear implantation or stapedotomy. The index test, usually biomarker detection, followed the reference standard. Assuming the definite presence of a connection between inner and middle ear during the surgery, separate data synthesis will be performed.

No assessment of meta-bias is planned

### 2.8 Assessment of confidence in cumulative evidence

Strength of evidence and clinical practice recommendations will be evaluated and presented using the GRADE (Grading of Recommendations, Assessment, Development, and Evaluations) framework (51). Evidence strength will be assessed as high (“We are very confident that the true effect lies close to that of the estimate of the effect”), moderate (“We are moderately confident in the effect estimate: The true effect is likely to be close to the estimate of the effect, but there is a possibility that it is substantially different”), low (“Our confidence in the effect estimate is limited: The true effect may be substantially different from the estimate of the effect”), or very low (“We have very little confidence in the effect estimate: The true effect is likely to be substantially different from the estimate of the effect”).

## 3 Amendments

If we need to amend this protocol, we will give the date of each amendment, describe the change and give the rationale in this section. Changes will not be incorporated into the protocol.

## 4 Reporting the review

The systematic review will be reported in accordance with Preferred Reporting Items for a Systematic Review and Meta-analysis of Diagnostic Test Accuracy Studies: The PRISMA-DTA Statement (46). A flowchart will be used to present the flow of study selection, screening, and inclusion, as well as any relevant findings related to the diagnostic accuracy of tests for PLF.
