# Supplementary material for: Accuracy of diagnostic tests for perilymphatic fistula: protocol for a systematic review
Source: Front Neurol. 2024 Nov 18;15:1500780. doi: 10.3389/fneur.2024.1500780 (PMC11609218; doi:10.3389/fneur.2024.1500780)
Supplement: Supplementary file 2 [file Table_2.docx]

**Supplement 2. Search strategy for the systematic review of perilymphatic fistula diagnostic tests accuracy.**

**Ovid MEDLINE(R) ALL <1946 to date>**

1 labyrinthine fluids/ or perilymph/

2 ((labyrinth* adj3 fluid*) or perilymph*).mp.

3 1 or 2

4 fistula/

5 (fistula* or leak*).mp.

6 4 or 5

7 3 and 6

8 fissula ante fenestram.mp.

9 Round Window, Ear/

10 (round window* or (cochlea* adj (window* or fenestra))).mp.

11 9 or 10

12 "wounds and injuries"/ or rupture/

13 (wound* or injur* or rupture*).mp.

14 12 or 13

15 11 and 14

16 7 or 8 or 15

**Embase Classic+Embase <1947 to date>**

1 perilymph/

2 ((labyrinth* adj3 fluid*) or perilymph*).mp.

3 1 or 2

4 fistula/

5 (fistula* or leak*).mp.

6 4 or 5

7 3 and 6

8 perilymph fistula/

9 fissula ante fenestram.mp.

10 cochlea fenestra/

11 (round window* or (cochlea* adj (window* or fenestra))).mp.

12 10 or 11

13 rupture/ or injury/

14 (wound* or injur* or rupture*).mp.

15 13 or 14

16 12 and 15

17 7 or 8 or 9 or 16

18 limit 17 to conference abstracts

19 17 not 18

**Cochrane Library**

#1 MeSH descriptor: [Labyrinthine Fluids] this term only

#2 MeSH descriptor: [Perilymph] explode all trees

#3 ((labyrinth* NEAR/3 fluid*) OR perilymph*):ti,ab,kw

#4 {OR #1-#3}

#5 MeSH descriptor: [Fistula] this term only

#6 (fistula* OR leak*):ti,ab,kw

#7 #5 OR #6

#8 #4 AND #7

#9 (fissula ante fenestram):ti,ab,kw

#10 MeSH descriptor: [Round Window, Ear] this term only

#11 ((round NEXT window*) or (cochlea* NEAR/2 (window* or fenestra))):ti,ab,kw

#12 #10 OR #11

#13 MeSH descriptor: [Wounds and Injuries] this term only

#14 MeSH descriptor: [Rupture] this term only

#15 (wound* OR injur* OR rupture*):ti,ab,kw

#16 {OR #13-#15}

#17 #12 AND #16

#18 #8 OR #9 OR #17

**Web of Science**

Topic:

(((labyrinth* NEAR/3 fluid*) or perilymph*) AND (fistula* OR leak*)) OR "fissula ante fenestram" OR ((round window* OR (cochlea* NEAR/1 (window* OR fenestra))) AND (wound* OR injur* OR rupture*))
