# Supplementary material for: Accuracy of diagnostic tests for perilymphatic fistula: protocol for a systematic review
Source: Front Neurol. 2024 Nov 18;15:1500780. doi: 10.3389/fneur.2024.1500780 (PMC11609218; doi:10.3389/fneur.2024.1500780)
Supplement: Supplementary file 3 [file Table_3.docx]

**Supplement 3. QUADAS-2 Risk of Bias and Applicability Judgments.** Reprinted from Whiting PF et al. Ann Intern Med. 2011;155(8):529-36. Available: <https://www.acpjournals.org/doi/10.7326/0003-4819-155-8-201110180-00009>. Reprinted with permission from Penny F. Whiting, PhD, School of Social and Community Medicine, Bristol, United Kingdom)

**The following table summarizes QUADAS-2 and lists all signalling, risk of bias, and applicability rating questions.**

| Domain | Patient selection | Index test | Reference standard | Flow and Timing |
| --- | --- | --- | --- | --- |
| Description | Describe methods of patient selection: Describe included patients (prior testing, presentation, intended use of index test and setting): | Describe the index test and how it was conducted and interpreted: | Describe the reference standard and how it was conducted and interpreted: | Describe any patients who did not receive the index test(s) and/or reference standard or who were excluded from the 2x2 table (refer to flow diagram): Describe the time interval and any interventions between index test(s) and reference standard: |
| Signalling questions: Yes/no/unclear | Was a consecutive or random sample of patients enrolled? | Were the index test results interpreted without knowledge of the results of the reference standard? | Is the reference standard likely to correctly classify the target condition? | Was there an appropriate interval between index test(s) and reference standard? |
|  | Was a case-control design avoided? | If a threshold was used, was it pre-specified? | Were the reference standard results interpreted without knowledge of the results of the index test? | Did all patients receive a reference standard? |
|  | Did the study avoid inappropriate exclusions? |  |  | Did all patients receive the same reference standard? |
|  |  |  |  | Were all patients included in the analysis? |
| Risk of bias: High/low/unclear | Could the selection of patients have introduced bias? | Could the conduct or interpretation of the index test have introduced bias? | Could the reference standard, its conduct, or its interpretation have introduced bias? | Could the patient flow have introduced bias? |
| Concerns regarding applicability: High/low/unclear | Are there concerns that the included patients do not match the review question? | Are there concerns that the index test, its conduct, or interpretation differ from the review question? | Are there concerns that the target condition as defined by the reference standard does not match the review question? |  |
